# LQTS Gene LOVD Database

**DOI:** 10.1002/humu.21341

**Published:** 2010-11

**Authors:** Tao Zhang, Arthur Moss, Peikuan Cong, Min Pan, Bingxi Chang, Liangrong Zheng, Quan Fang, Wojciech Zareba, Jennifer Robinson, Changsong Lin, Zhongxiang Li, Junfang Wei, Qiang Zeng, Ming Qi, Long QT International Registry Investigators, HVP-China Investigators

**Affiliations:** 1 James D. Watson Institute of Genome Sciences, College of Life Sciences, Zhejiang UniversityHangzhou, Zhejiang, China; 2 Center for Genetic and Genomic Medicine, Zhejiang University School of Medicine First Affiliated HospitalHangzhou, Zhejiang, China; 3 Department of Medicine(Cardiology), University of RochesterRochester, New York, USA; 4 Peking Union Medical College HospitalBeijing, China; 5 Department of Cardiology, Zhejiang University School of Medicine First Affiliated HospitalHangzhou, Zhejiang, China; 6 Zhejiang Academy of Medical SciencesHangzhou, Zhejiang, China; 7 Division of Sport Medicine, College of Education, Zhejiang UniversityHangzhou, Zhejiang, China; 8 General Hospital of PLABeijing, China; 9 Department of Pathology and Laboratory Medicine, University of RochesterRochester, New York, USA

**Keywords:** Long QT Syndrome, Arrhythmia, LOVD, Mutation database

## Abstract

The Long QT Syndrome (LQTS) is a group of genetically heterogeneous disorders that predisposes young individuals to ventricular arrhythmias and sudden death. LQTS is mainly caused by mutations in genes encoding subunits of cardiac ion channels (KCNQ1, KCNH2, SCN5A, KCNE1, and KCNE2). Many other genes involved in LQTS have been described recently (KCNJ2, AKAP9, ANK2, CACNA1C, SCNA4B, SNTA1, and CAV3). We created an online database (http://www.genomed.org/LOVD/introduction.html) that provides information on variants in LQTS-associated genes. As of February 2010, the database contains 1738 unique variants in 12 genes. A total of 950 variants are considered pathogenic, 265 are possible pathogenic, 131 are unknown/unclassified, and 292 have no known pathogenicity. In addition to these mutations collected from published literature, we also submitted information on gene variants, including one possible novel pathogenic mutation in the *KCNH2* splice site found in ten Chinese families with documented arrhythmias. The remote user is able to search the data and is encouraged to submit new mutations into the database. The LQTS database will become a powerful tool for both researchers and clinicians. © 2010 Wiley-Liss, Inc.

## INTRODUCTION

Long QT Syndrome (LQTS) is a familial disorder characterized by prolongation of the QT-interval and a high incidence of sudden cardiac death mostly at a young age. Two phenotypic variants have been described: i) the more common autosomal dominant Romano-Ward syndrome (Romano, et al., 1963; Ward, 1964), and ii) the less common autosomal recessive Jervell and Lange-Nielsen syndrome, which is associated with sensorineural deafness (Jervell and Lange-Nielsen, 1957). The hereditary LQTS is a genetic channelopathy with variable penetrance that is associated with increased propensity for polymorphic ventricular tachyarrhythmias, particularly torsades de pointes, leading to syncope, seizures and sudden death in young patients with normal cardiac morphology. The disease is relatively infrequent, with variable prevalence estimated from 1:2000 to 1:5000 (Goldenberg, et al., 2008; Schwartz, et al., 2009).

QT prolongation is the hallmark of LQTS, and it may form via one of two pathways: reduction in the outward potassium current during phase 3 of the action potential (“loss of function”) or an augmented late entry of sodium or calcium ions into the cardiac myocytes (“gain of function”) (Goldenberg, et al., 2008; Moss and Kass, 2005). In 1995, Curran et al. first found LQTS caused by KCNH2 gene mutations (Curran, et al., 1995). The rapidly activating potassium repolarization channel mutation (KCNH2; LQT2) results in a reduction in IKr current. Wang et al. reported *SCN5A* mutations associated with congenital cardiac arrhythmia and LQTS (Wang, et al., 1995). This sodium channel mutation (SCN5A; LQT3) results in an increase in late INa current. In 1996, the KCNQ1 gene was identified as a cause of LQTS (Wang, et al., 1996). The slowly activating potassium repolarization channel mutation (KCNQ1; LQT1) results in a reduction in IKs current. LQTS has also been identified infrequently in patients with mutations involving the auxiliary β-subunits of KCNQ1 (mink, KCNE1; LQT5) (Splawski, et al., 1997) and of KCNH2 (MiRP1, KCNE2; LQT6 (Abbott, et al., 1999), respectively. Mutations in five genes (KCNQ1, KCNH2, SCN5A, KCNE1, and KCNE2) account for approximately 72% of clinically definite LQTS (Napolitano, et al., 2005). Mutations in three other ion-channel genes have been identified in some LQTS families: i) mutation of the KCNJ2 gene results in a reduction in Kir2.1 current, long QT interval and skeletal abnormalities (Andersen-Tawil's syndrome) (LQT7; Plaster, et al., 2001); ii) mutation in the CACNA1C gene results in an increase in Cav1.2 current, QT prolongation, and multiorgan dysfunction, including webbing of fingers and toes, congenital heart disease, immune deficiency, intermittent hypoglycemia, cognitive abnormalities, and autism (Timothy syndrome) (LQT8; Splawski, et al., 2004); iii) mutation in the SCN4B gene causes an increase in late sodium current (LQT10; Medeiros-Domingo, et al., 2007). A summary of LQT1-12 genotypes, their affected ion-channel currents, and their variant distribution found in our database is presented in Table 1.

**Table 1:** Summary of 12 LQTS-associated genes

Phenotype	LQT1	LQT2	LQT3	LQT4	LQT5	LQT6	LQT7	LQT8	LQT9	LQT10	LQT11	LQT12
**Gene**	*KCNQ1*	*KCNH2*	*SCN5A*	*ANK-2*	*KCNE1*	*KCNE2*	*KCNJ2*	*CACNA1C*	*CAV3*	*SCN4B*	*AKAP9*	*SNTA1*
**Chromosome**	11p15.5	7q35-36	3p21	4q25-27	21q22	21q22	17q23.1-24.2	12p13.3	3p25	11q23.3	7q21-22	20q11.2
**OMIM#**	607542	152427	600163	106410	176261	603796	600681	114205	601253	608256	604001	601017
**No pathogenicity**	75	61	84	14	15	9	5	2	8	2	13	4
**Possible pathogenicity**	56	67	105	9	19	6	2	0	0	0	1	0
**Pathogenicity**	333	433	189	7	16	13	45	5	6	1	1	1
**Unknown pathogenicity**	7	29	24	30	1	0	1	0	0	3	32	4
**Total unique mutations**	471	590	402	60	51	28	53	7	14	6	47	9

Advances in molecular genetics have helped reveal a number of genes that may give rise to LQTS. In addition to these eight ion channel genes described above, mutations in non-ion channel genes can also affect ion-channel currents through direct or indirect interaction with the ion channel complexes. Thus far, four non-ion channel LQTS-susceptibility genes have been discovered: (1) the ankyrin-B gene, which encodes a protein that functions as a cytoskeletal membrane adapter and is involved with the sodium pump, the sodium/calcium exchanger, and the inositol-1,4,5-triphosphate receptors, and can cause LQT4 when mutated (Mohler, et al., 2003); (2) caveolin-3, which alters gating kinetics in the cardiac sodium channel, and if mutated may result in an increase in sustained late sodium current (Nav1.5; LQT9)(Cronk, et al., 2007; Vatta, et al., 2006); (3) AKAP9 (LQT11), mutation of which reduces the interaction between KCNQ1 and AKAP9 (Yotiao), reduces the cAMP-induced phosphorylation of the channel, eliminates the functional response of the IKs channel to cAMP, and prolongs the QT interval (Chen, et al., 2007); (4) SNTA1 (LQT12), which when mutated increases direct nitrosylation of SCN5A and results in augmentation of late sodium current (Ueda, et al., 2008). Despite this progress in uncovering the genes responsible for LQTS, roughly 25% of patients with clinical LQTS are negative for mutations in the twelve LQTS-associated genes, indicating that more genetic abnormalities remain to be identified.

The variants that have been found in the identified LQTS-associated genes are of different types. To date, hundreds of nonsynonymous (amino-acid-altering, missense, nonsense, and frameshift) mutations and splice-site altering mutations have been found in these twelve LQTS-susceptibility genes. Out of 1738 published or reported unique variants, mutations in KCNQ1, KCNH2 and SCN5A genes account for almost 85% of total LQTS-associated mutations collected in our database (See Table 1). However, discerning the clinical relevance and pathogenicity of individual mutations is still a challenge. Classification of LQTS-associated gene mutations is generally based on the following several criteria: 1) the electrophysiological abnormality of the ion-channel caused by the mutation; 2) the structure of the protein formed by frameshift, splice-site or nonsense mutations; 3) amino acid changes in the conserved domains of a gene due to missense mutation; 4) failure of protein trafficking due to mutation; 5) the relative frequency of the mutation in healthy individuals.

Two earlier LQTS-variant databases have been set up, one of which collected 232 mutations and 27 polymorphisms through 2003 (including KCNQ1, KCNH2, SCN5A, KCNE1 and KCNE2) (http://www.ssi.dk/graphics/html/lqtsdb/lqtsdb.htm); the other database collected over 798 mutations and 122 polymorphisms through 2007 (including LQT1-LQT9) (http://www.fsm.it/cardmoc/). Both databases have their own unique features, though neither has been updated. Clinicians and researchers need more comprehensive and timely information about genes associated with LQTS. Thus, we here established this LQTS-variant database (http://www.genomed.org/LOVD/LQTs/home.php) to allow researchers and physicians access to comprehensive and current mutation information.

## DATABASE STRUCTURE

### Data Collection and Submission

The bulk of the data on gene variants is derived from published papers and the NCBI SNP database; we also included our own data on the mutations found in ten Chinese families with arrhythmias. For the compiled mutations extracted from the literature, we searched Entrez PubMed (http://www.ncbi.nlm.nih.gov/sites/entrez) using “Long QT syndrome”, “Sudden unexplained cardiac death”, and the names and abbreviations of genes published as related to LQTS (KCNQ1, KCNH2, SCN5A, KCNE1, KCNE2, KCNJ2, AKAP9, ANK2, CACNA1C, SCNA4B, SNTA1, and CAV3) as key words. English and Chinese papers matching these search results were collected, as well as papers in other languages that had English abstracts. From the selected papers and abstracts, we compiled the mutations, including the details of DNA and amino acid changes, and judged the classification of pathogenicity reported by the authors. In general, silent mutations and mutations reported in healthy controls were designated as “not known pathogenicity” (Ackerman, et al., 2003; Gouas, et al., 2005; Jongbloed, et al., 2002). Missense mutations found as a result of large screenings of patients with LQTS or Sudden Infant Death Syndrome, and which lack sufficient data to support their pathogenicity, were categorized as “possible pathogenicity” (Jongbloed, et al., 2002; Kapplinger, et al., 2009; Napolitano, et al., 2005; Splawski, et al., 2000; Tester, et al., 2005). The mutation names comply with the accepted guidelines proposed by the Human Genome Variation Society(HGVS)(http://www.hgvs.org/mutnomen) (den Dunnen and Antonarakis, 2000). We also searched the NCBI SNP database (http://www.ncbi.nlm.nih.gov/SNP) and included these SNPs in our database. However, we uniformly classified the pathogenicity of these SNPs as “unknown”, except for those SNPs already described in other published papers.

Because of the large volume of LQTS-related articles, we organized an Human Virome Project(HVP) student club to recruit volunteers interested in genetic and genomic medicine at Zhejiang University. After being trained, these volunteers were divided into several groups to upload the data in the Leiden Open Variation Database (LOVD) format (Fokkema, et al., 2005). Once submitted, the uploaded data were checked by curators before being released for access by the public.

We have also screened 10 Chinese families with various clinical arrhythmias, including 3 with LQTS, 2 with Brugada syndrome, 3 with sudden death syndrome, and 2 with Sick Sinus syndrome for mutations in KCNQ1, KCNH2, SCN5A, KCNE1 and KCNE2. Several common polymorphisms were identified, including p.Ser38Gly in KCNE1 and p.Arg1 193Gln in SCN5A. Interestingly, a novel splice mutation c.2690A>C in SCN5A was discovered in a Sick Sinus syndrome patient with compound mutations c.1141-3C>A (homozygous) and p.His558Arg in SCN5A. This novel mutation has not been previously reported and we categorized the mutation as “possible pathogenicity”; a functional analysis is still ongoing. Two pathogenic mutations were found in two families: p.81_82delIleAlaGln in KCNH2 and p.Asp1275Asn in SCN5A, which have both been previously described. All this data have been included in the database.

### Database Structure

The database is based on the Leiden Open Variation Database system, which is a web-based database format designed to collect and display DNA variants in specific genes (Fokkema, et al., 2005).

**Figure 1:**
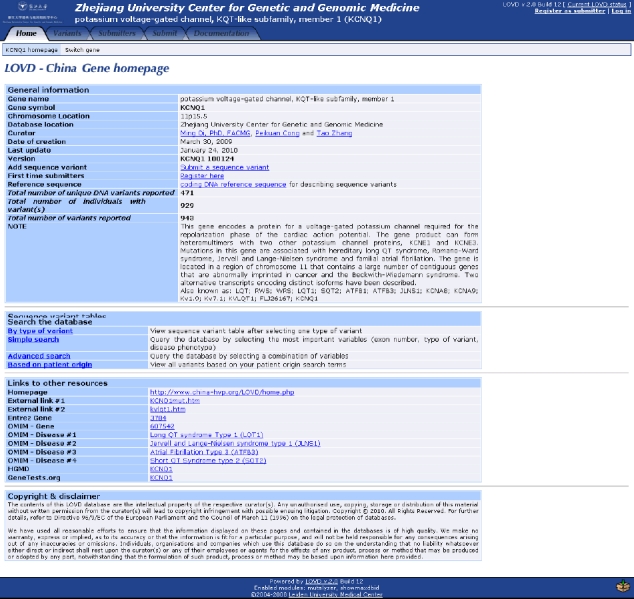
Homepage of the KCNQ1 database. The main function menu, shown on the left side, contains the four options provided for the users, which is available at the website http://www.genomed.org/LOVD/LQTs/home.php?select_db=KCNQ1.

Our database website is http://www.genomed.org/LOVD/introduction.html. On the homepage is a simple table, with the left row showing the 12 total LQTS-associated genes. Each gene links to its own home database (see Figure 1). For example, the web page for the KCNQ1 variant database consists of four sections: general information, sequence variant tables, database search, and links to other resources. Each section provides useful information through a user-friendly interface. At the top of the web page are function buttons designated “Home”, “Variants”, “Submitters”, “Submit” and “Document”. The remote user is able to search the data and is encouraged to submit new mutations into the database after registering as a submitter.

### Database Content

**Figure 2:**
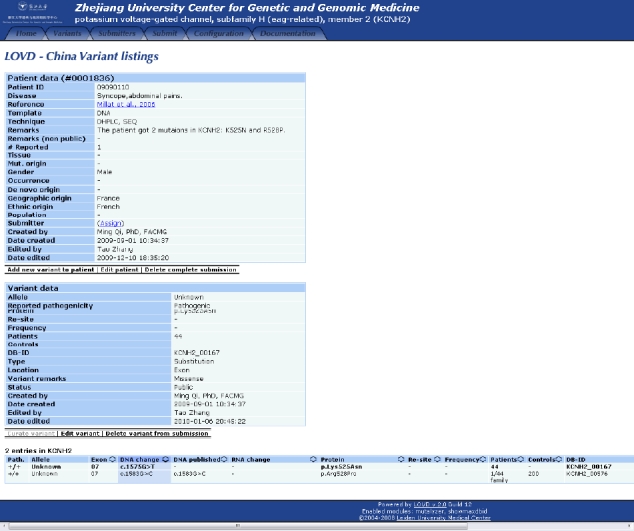
Details of mutation KCNH2: p.Lys525Asn. In addition to the p.Lys525Asn mutation, the patient carries another mutation KCNH2: p.Arg528Pro. The page also contains the articles reporting the two variants, with a link to their corresponding Pubmed entries. This page is available at http://www.genomed.org/LOVD/LQTs/variants.php?select_db=KCNH2&action=view&view=0001836%2C0000205%2C0

In the KCNQ1 variant home database, there are 943 total variants reported (see Figure 1), which are separated into 471 unique DNA variants. Each entry contains two categories of information: patient data and variant data (see Figure 2). The patient data contains the following items: disease, reference, template, technique, remarks, mutation origin, gender, occurrence, de novo origin, geographic origin, ethnic origin and population. The section for variant data is likewise separated into subcategories as follows: allele, reported pathogenicity, concluded pathogenicity, exon, DNA change, DNA published, RNA change, protein, restriction site, frequency, patients, control, DB-ID, type, location and variant remarks.

The data for each gene is based on the published literature, but many published articles do not provide all the details that are available for input in the database, as described above. For example, many papers only describe the amino acid changes that result from DNA mutations. Thus, it is left to the database inputter to check the wildtype DNA sequence, fill in the “DNA change” item as appropriate for that amino acid mutation, and then to mark the “DNA published” item as “No”. For complicated amino acid changes, such as frameshifts, we have opted to add “c.?” in the “DNA change” category if the DNA sequence is not already published (Makita, et al., 2007; Mank-Seymour, et al., 2006; Meregalli, et al., 2009; Moss, et al., 2002; Struijk, et al., 2006; Westenskow, et al., 2004).

One gene in particular, ANK-2, does not have a consensus DNA sequence. For this gene, which is associated with LQT4 syndrome, we have downloaded a reference sequence from NCBI (NM00127493.1). Some published articles, however, use a reference sequence different from our selected reference sequence; in these cases, we respect the authors' choice by submitting the DNA change as described in those papers (Mank-Seymour, et al., 2006; Mohler, et al., 2007; Mohler, et al., 2004; Sherman, et al., 2005).

## DISCUSSION

We have set up a publicly accessible online database for variants in genes associated with LQTS. The database contains the most comprehensive variant data available from the published literature, including the entire corpus of Chinese literature on the subject. The database will not only assist clinical geneticists in counseling families found to have a variant of these genes, but will also aid genetic scientists investigating the function of the mutations, which should reduce the time spent searching the literature and help to predict the possible pathogenetic nature of the variant.

The 12 genes contained in the database appear not only to be associated with LQTS, but also with other syndromes. Mutations in the SCN5A gene, for instance, are also found in patients with Brugada syndrome, cardiac conduction defects, sudden infant death syndrome, arrhythmogenic right ventricular cardiomyopathy, and sick sinus syndrome (Makita, et al., 2005; Miyoshi, et al., 2005; Priori, et al., 2002; Priori, et al., 2000). Even within a single family, the same variant may present different phenotypes in different family members (Bezzina, et al., 1999; Smits, et al., 2005). Our database includes this valuable data by listing the other associated diseases in the patient data section. Moreover, patients carrying two or three mutations in these 12 genes (especially in the KCNQ1, KCNH2, SCN5A, KCNE1 and KCNE2 genes) may have severe clinical symptoms (Shim, et al., 2005; Westenskow, et al., 2004; Ning, et al., 2003b). We also present this information in our database under the rubric for “Remarks” (Figure 2).

**Figure 3:**
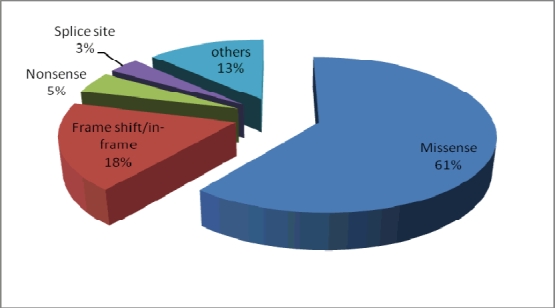
Pie chart showing the proprotion of KCNQ1,KCNH2,SCN5a,KCNE1 and KCNE2 mutation types. “Others” includes mutations in introns, the 5′UTR and 3′ UTR, and silent mutations.

The distribution of mutations is not always random. Marjamaa et al revealed four founder mutations which constitute up to 70% of the known genetic spectrum of LQTS in 6,334 Finnish subjects (Marjamaa, et al., 2009). The four founder mutations are KCNQ1 p.Gly589Asp, KCNQ1 C.1033-2A>G (IVS7-2A>G), KCNH2 p.Leu552Ser and KCNH2 p.Arg176Trp, which have a prevalence estimate of 0.4% (95% CI 0.3%–0.6%) in the Finnish population (Marjamaa, et al., 2009). A comprehensive mutational analysis involving 744 apparently healthy individuals from four race/ethnicity groups (black, white, Asian and Hispanic) revealed that even the common polymorphisms were not equally distributed; p.Lys897Thr-KCNH2 was more common in whites, while p.Pro448Arg-KCNQ1 was almost absent in whites and more often in Asians (Ackerman, et al., 2003). The proportion of the different types of mutations from the five major LQTS-associated genes (KCNQ1, KCNH2, SCN5A, KCNE1 and KCNE2) is represented in a pie chart demonstrating that missense and frameshift mutations account for 79% (Figure 3).

The 12 LQTS susceptible genes have been shown to account for about 36% to 72% of identified variants in LQTS patients (Berge, et al., 2008; Kapplinger, et al., 2009; Napolitano, et al., 2005; Splawski, et al., 2000; Tester, et al., 2005; Ning, et al., 2003a). There remain 30% or more LQTS patients without a documented pathogenic variation in these genes. There may be other genes involved in these patients, or the mutations may be in the introns or other so-called junk sequences of the 12 known genes, which can affect their expression or translation procession. Crotti et al identified a c.23 99-28A>G (IVS9-28A/G) mutation in KCHN2 that disrupted the acceptor splice site definition by affecting the branch point (BP) sequence and thereby promoting intron retention (Crotti, et al., 2009). In other LQTS patients, a large segment duplication or deletion has been identified, which were not easily screened for by current polymerase chain reaction-based exon-scanning methods (Eddy, et al., 2008; Koopmann, et al., 2006).

Our database provides the most complete and universal format published variants for LQTS, although further investigation will likely yield more data. As new variant are identified, we will update the database with the help of remote users and scholars, who may submit their own variants.
